# Climate change, air pollution and noncommunicable diseases

**DOI:** 10.2471/BLT.18.224295

**Published:** 2018-12-19

**Authors:** Diarmid Campbell-Lendrum, Annette Prüss-Ustün

**Affiliations:** a Department of Public Health, Environmental and Social Determinants of Health, World Health Organization, avenue Appia 20, 1211 Geneva 27, Switzerland.

The World Health Organization (WHO) has identified climate change as one of the greatest health threats of the 21st century, and air pollution as the single largest environmental health risk.^1^ At the same time, noncommunicable diseases constitute the largest and fastest growing global health burden, with treatment costs placing a massive strain on government and individual resources.

The scaling up of international commitment on noncommunicable diseases over the past decade had initially focused on four risk factors: tobacco use, the harmful use of alcohol, unhealthy diet and physical inactivity. Exposure to each of these risks has a strong element of personal choice, with the responsibility often placed on individual rather than on broader societal responses. However, these risks are also strongly affected by social determinants, including commodity prices, production methods, marketing and social norms, and in the case of activity levels, the physical environment. A range of other risk factors for noncommunicable diseases are even more strongly linked to environmental exposures – and to climate change. 

Therefore, together, climate change, air pollution and noncommunicable diseases represent one of the most serious threats to global health. Many of the same development patterns that lead to high reliance on fossil fuels, as well as policies and technological choices that are driving climate change (such as polluting transport and energy choices) are also worsening air pollution and other environmental exposures. These exposures have a direct and strong influence on the prevalence of noncommunicable diseases. The most obvious is air pollution. Indoor and outdoor air pollution is responsible for an estimated 7 million deaths a year^2^ and comes second to tobacco as a risk factor for noncommunicable diseases. Air pollution has therefore been identified as the fifth major risk factor in the latest political declaration of the United Nations General Assembly on the prevention and control of noncommunicable diseases.

The effects of air pollution on health show that noncommunicable diseases are not exclusively due to lifestyle or personal choices, as is commonly perceived. Recommendations to stay indoors, avoid walking along particularly polluted streets or to wear facemasks during episodes of high exposure to air pollution illustrate the inadequacy of individual responses to a broad and serious problem.

The ultimate causes of air pollution, and therefore of a large proportion of the noncommunicable disease burden, are the energy sources that currently drive our transport, electricity generation, industry and food production systems.

The connection between the sources of local air pollution and the emissions that drive climate change is very clear. Estimations show that approximately 25% of urban ambient air pollution from fine particulate matter (PM_2.5_) is contributed by traffic, 15% by industrial activities including electricity generation, 20% by domestic fuel burning (with a remaining 22% from unspecified sources of human origin and 18% from natural sources).^3^ Exposure to indoor air pollution is mostly due to the use of solid fuels for cooking in low-income households.^4^ Such exposure causes almost 4 million deaths a year, of which almost 3 million are due to noncommunicable diseases such as lung cancer, chronic obstructive pulmonary disease, ischaemic heart disease and stroke.

For comparison, the International Panel on Climate Change estimates that global greenhouse gas emissions are caused by transport (14%), energy; including generation of electricity and heat (35%), industry (21%), buildings (6%) and agriculture and land use change (24%).^5^ The sources of climate change and air pollution, and therefore a large part of the noncommunicable disease burden, are broadly the same: polluting energy systems.

Some of the same pollutants contribute both to climate change and local ambient and household air pollution. Black carbon, produced by inefficient combustion in sources such as cookstoves and diesel engines, is the second greatest contributor to global warming after carbon dioxide.^5^ Black carbon is also a significant contributor (between 5% and 15%) of urban exposure to PM_2.5_. The second largest contributor to global warming is methane, which reacts with other pollutants to form ozone and is responsible for 230  000 chronic respiratory disease deaths globally each year.^6^ Both of these pollutants are short-lived in the atmosphere, meaning that targeting them for removal would have immediate beneficial effects on both climate change and noncommunicable diseases, such as stroke and deaths from cardiovascular disease. A set of 16 practical interventions, from replacing polluting cookstoves with cleaner household energy solutions, to replacing the most polluting diesel fuels and engines with less polluting ones, would prevent approximately 0.5°C of global warming, and save some 2.5 million lives a year by 2050.^7^

Other opportunities are available: improving energy efficiency and insulation in houses in temperate climates, therefore reducing mortality from respiratory and cardiovascular deaths in winter; transitioning from polluting solid fuels to clean and sustainable energy in low-income households, therefore reducing deaths from indoor air pollution; adopting reliable renewable energy in health-care facilities not connected to electricity grids, therefore allowing refrigeration of medical supplies and lighting for essential services.^8^

In the transport sector, an accelerated transition from diesel and petrol engines to electric powered vehicles would contribute to reducing emissions of local air pollutants and greenhouse gas. Much greater health gains, however, would result from replacing short urban car journeys with walking and cycling, due to increases in physical activity. Modelling based on systematic reviews of the health effects of increased active travel indicated that reductions in air pollution due to increased active travel could prevent 21 premature deaths per million population per year in London, and 99 per million population per year in Delhi. The gains of physical activity are expected to be even greater. As one third of adults and four-fifths of adolescents do not reach the activity levels recommended by WHO, it is estimated that the disability-adjusted life years saved from increased physical activity due to active travel policies could be 37 to 74 times higher than those saved from the reductions in air pollution.^9^

1) Similar considerations apply to agriculture and land use, responsible for approximately a quarter of greenhouse gas emissions. More sustainable agricultural production measures, such as reducing open burning of agricultural land, would help mitigate climate change and reduce air pollution in some regions. However, even greater gains may be obtained by reducing human consumption of meat and by reducing food waste. Even though meat and dairy make a relatively small contribution to overall human energy intake, around 60–80% of the greenhouse gas emissions from agriculture come from the livestock sector, which also has a range of additional environmental impacts, from deforestation to water contamination and degradation of topsoil, which are increasing with the growing demand from emerging economies.^10^ Reducing meat intake in high-consuming populations can therefore be expected to significantly reduce environmental impacts. Modelling the effect of potential strategies to meet national commitments to reduce greenhouse gas emissions from the agricultural sector in the United Kingdom of Great Britain and Northern Ireland look promising. For example, reducing livestock production and consumption of red meat, indicated that these strategies could be expected to result in a 15% reduction in disease burden due to reduced consumption of saturated fats and associated heart disease.

Even more wide-ranging effects could be brought about by fiscal policy. Studies by the International Monetary Fund show that the global production and consumption of highly polluting fuels is indirectly subsidized with over 5 trillion United States dollars (US$) a year, which is more than all governments around the world spend on health care.^11^ This de facto subsidy exists because the health and climate damages that they cause are not reflected in fuel prices. Approximately half of these US$ 5 trillion are from the uncosted health impacts of air pollution, mainly from coal. Increasing the price of fuels, consistent with the damage that they cause to health and to the global climate system would remove this unfair advantage. Such increase would be expected to bring about a shift to cleaner energy sources that would reduce air pollution deaths by half, decrease global carbon dioxide emissions by approximately 20%, and generate about US$ 3 trillion a year in revenue.^12^ This revenue could be directed to socially beneficial investments, for example to universal health coverage and education.

Financial reasons should no longer constitute an obstacle to bring about these changes. In many cases, cleaner and greener technologies are now cheaper than polluting alternatives, particularly if the health gains are accounted for. Implementing such alternatives needs political will and a shift in mindsets. Fiscal, energy or transport policies need to consider the externalities on health to become tools that advance overall sustainable development.

Due to of the connections between environmental degradation and the human and financial costs of noncommunicable diseases, the health sector should have a say in related policy debates.

## References

[1] Ambient air pollution: a global assessment of exposure and burden of disease. Geneva: World Health Organization; 2016. Available from http://www.who.int/phe/publications/air-pollution-global-assessment/en/ [cited 2018 Oct 11]

[2] Burden of disease from the joint effects of household and ambient air pollution for 2016. Geneva: World Health Organization; 2016. Available from: http://www.who.int/airpollution/data/AP_joint_effect_BoD_results_May2018.pdf?ua=1 [cited 2018 Nov 27].

[3] Karagulian F, Belis CA, Dora CG, Prüss-Ustün AM, Bonjour S, Adair-Rohani H, et al. Contributions to cities’ ambient particulate matter (PM): A systematic review of local source contributions at global level. Atmos Environ. 2015;120:475–83. 10.1016/j.atmosenv.2015.08.087

[4] Household air pollution and health [internet]. Geneva: World Health Organization; 2018. Available from: http://www.who.int/en/news-room/fact-sheets/detail/household-air-pollution-and-health [cited 2018 Nov 27].

[5] International Panel on Climate Change. Summary for policymakers. In: Climate change 2014: mitigation of climate change. Contribution of working group III to the fifth assessment report of the intergovernmental panel on climate change. Cambridge: Cambridge University Press; 2014. Available from: https://www.ipcc.ch/report/ar5/wg3/ [cited 2018 Oct 5].

[6] Global health data exchange. Seattle: Institute for Health Metrics and Evaluation; 2018. Available from: http://ghdx.healthdata.org/gbd-results-tool [cited 2018 Oct 5].

[7] Reducing global health risks through mitigation of short-lived climate pollutants: scoping report for policy makers. Geneva: Climate and Clean Air Coalition, World Health Organization; 2015. Available from: http://apps.who.int/iris/bitstream/handle/10665/189524/9789241565080_eng.pdf?sequence=1 [cited 2018 Oct 5].

[8] Haines A, McMichael AJ, Smith KR, Roberts I, Woodcock J, Markandya A, et al. Public health benefits of strategies to reduce greenhouse-gas emissions: overview and implications for policy makers. Lancet. 2009 12 19;374(9707):2104–14. 10.1016/S0140-6736(09)61759-1 19942281

[9] Woodcock J, Givoni M, Morgan AS. Health impact modelling of active travel visions for England and Wales using an integrated transport and health impact modelling tool (ITHIM). PLoS One. 2013;8(1):e51462. 10.1371/journal.pone.0051462 23326315 PMC3541403

[10] Steinfeld H, Gerber P, Wassenaar T, Castel V, Rosales M, de Haan C. Livestock’s long shadow: environmental issues and options. Rome: Food and Agriculture Organization of the United Nations; 2006.

[11] Coady D, Parry I, Sears L, Shang B. How large are global energy subsidies? WP/15/105. IMF working paper. Washington, DC: International Monetary Fund; 2016 Available from: https://www.imf.org/external/pubs/ft/wp/2015/wp15105.pdf[cited 2018 Oct 5].

[12] Parry I, Veung C, Heine D. How much carbon pricing is in countries’ own interests? The critical role of co-benefits. Washington, DC: International Monetary Fund; 2014 10.5089/9781498358279.001

